# Therapeutic Notes upon Convalescence

**Published:** 1887-09

**Authors:** 


### THERAPEUTIC NOTES UPON CONVALESCENCE.

E. J. L.

Appetite, does not return : Psor., Sulph.
— delayed, feverish, faint feeling : Cocc.
— cannot eat, everything tastes bitter : Puls.
— ravenous : Ars., Puls.
Chilliness, and sensitiveness to least draught : Selen.
Desire for eggs : Calc.
Despair of recovery : *Psor.*
Hemicrania : Ign.
Lower limbs feel as if paralyzed : Selen.
Marasmus : China.
Memory, loss of : Anac.
Periostitis of sacrum : Sil.
Prostration : Psor.
Recovery, slow : Chin.
— — with diarrhœa : Chin., Rheum.
— — protracted cases, with mild delirium, anxiety, and restlessness : Ars.
Relapse from overexertion : Rhus.
— from overexertion of body and mind : Cupr.
— from exertion of mind : Nux vom.
— from fright : Ign.
— from anger : Nux vom.
Rheumatic toothache : Rhod.
Rheumatism, obstinate : Colch.
Sexual desire, great : Aloe, Phos., Psor.
Unpleasant sensations, running downward : Guajac.
— — — upward : Fluor.-ac., Selen.
Vomiting, bilious : Chin.
— obstinate : Creos.
Weakening sweats, day and night : Psor.

